# Identification of small molecule inhibitors of the Lin28-mediated blockage of pre-let-7g processing†
†Electronic supplementary information (ESI) available: Additional control binding experiments, IC_50_ determining binding experiments, chemical structures and control Dicer processing experiments. See DOI: 10.1039/c6ob01945e



**DOI:** 10.1039/c6ob01945e

**Published:** 2016-10-04

**Authors:** Helen L. Lightfoot, Eric A. Miska, Shankar Balasubramanian

**Affiliations:** a Department of Chemistry , University of Cambridge , Lensfield Road , Cambridge , CB2 1EW , UK . Email: sb10031@cam.ac.uk ; Fax: +44 (0)1223 336913 ; Tel: +44 (0)1223 336347; b Wellcome Trust Cancer Research UK Gurdon Institute , University of Cambridge , Tennis Court Rd , Cambridge , CB2 1QN , UK . Email: eric.miska@gurdon.cam.ac.uk ; Fax: +44 (0)1223 334088 ; Tel: +44 (0)1223 767220; c Cancer Research UK , Cambridge Institute , Li Ka Shing Centre , Robinson Way , Cambridge , CB2 0RE , UK

## Abstract

The protein Lin28 and microRNA let-7 play critical roles in mammalian development and human disease. Lin28 inhibits let-7 biogenesis through direct interaction with let-7 precursors (pre-let-7). Accumulating evidence *in vitro* and *in vivo* suggests this interaction plays a dominant role in embryonic stem cell self-renewal and tumorigenesis. Thus the Lin28–let-7 interaction might be an attractive drug target, if not for the well-known difficulties in targeting protein–RNA interactions with drugs. The identification and development of suitable probe molecules to further elucidate therapeutic potential, as well as mechanistic details of this pathway will be valuable. We report the development and application of a biophysical high-throughput screening assay for the identification of small molecule inhibitors of the Lin28–pre-let-7 interaction. A library of pharmacologically active small molecules was screened and several small molecule inhibitors were identified and biochemically validated. Of these four validated inhibitors, two compounds successfully restored processing of pre-let-7g in the presence of Lin28, validating the concept. Thus, we have identified examples of small molecule inhibitors of the interaction between Lin28 and pre-let-7. This study provides a proof of concept for small molecule inhibitors that antagonise the effects of Lin28 and enhance processing of let-7 miRNA.

## Abstract 2

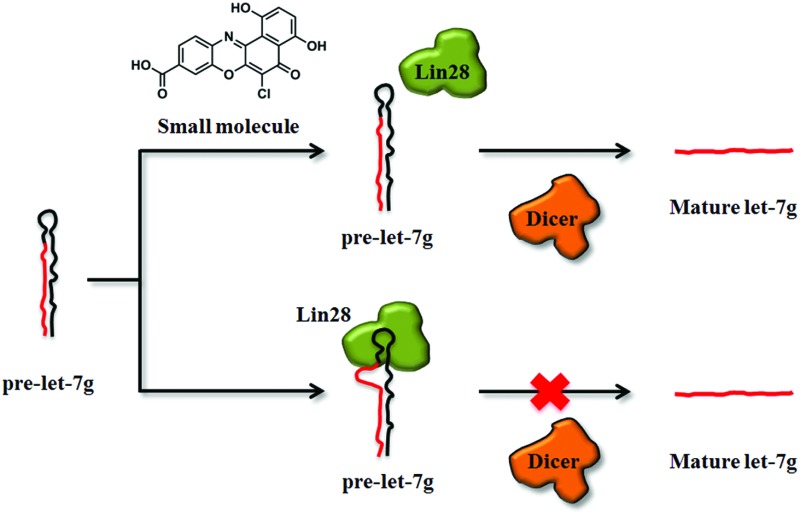
Small molecules enhance Dicer processing of a let-7 miRNA precursor through antagonization of the Lin28–pre-let-7 interaction.

## Introduction

Studies *in vitro* and *in vivo* have generated compelling data to support the role of the micro-ribonucleic acid (miRNA) let-7 (Lethal 7) as a *bona fide* tumour suppressor gene, consistent with its involvement in regulating cell proliferation and differentiation.^1–3^ The biogenesis of specific members of the let-7 family of miRNAs is inhibited by the pluripotency factor, human abnormal cell lineage protein 28 (Lin28), predominantly at the Dicer processing step, in both embryonic stem cells (ESC) and embryonic carcinoma cells.^4–6^ This inhibition is mediated by direct interactions between Lin28 and the let-7 precursor (pre-let-7),^7–9^ and has been suggested to be the result of a consequent combination of RNA structural changes,^10–12^ steric effects^6,10,11,13^ and uridylation.^14–17^ Lin28 assembles in a stepwise manner on pre-let-7 to form a stable multimeric complex.^10,13^ Observation of the lower stoichiometric Lin28–pre-let-7 complexes is dependent on the concentration of Lin28 and the presence of competitor RNAs.^10,13^ Mapping studies investigating complex formation using a variety of biophysical and biochemical methods^13^ coincide remarkably well with RNase protection studies reported by our group;^10^ here two motifs in the pre-let-7 terminal loop were required for Lin28 binding. Such interactions were responsible for destabilization of Watson–Crick base pairs within the terminal loop^10,13^ and consequently capable of inhibiting dicer processing of pre-let-7.^10^ Lin28 itself is involved in a variety of let-7-dependent and independent cellular processes; examples include cellular reprogramming,^18–23^ proliferation,^20,24^ skeletal myogenesis,^25^ glucose metabolism,^26^ neurogliogenesis^6,27^ and tumorigenesis.^28–31^ Lin28 is thought to act as an oncogene at least in part due to its role in the suppression of specific members of the let-7 family.^28,32–34^ For example, a terminal loop mutant of pre-let-7g and a loopmiR targeting the pre-let-7a-1 terminal loop, both capable of directing pre-let-7 away from a Lin28-mediated Dicer processing block, were shown to reverse Lin28-directed cellular transformation.^28,35^ These observations suggest that the LIN28–let-7 interaction might be an attractive target for conventional small molecule therapies; however, the well-known difficulties in targeting RNA–protein interactions with small molecules^30,31^ hamper validation of this hypothesis. Small molecule probes capable of restoring the levels of let-7 miRNAs through inhibition of the Lin28–pre-let-7 interaction would be powerful tools for assessment of its potential as a novel target in human disease, as well as for further elucidation of this pathway.

We describe the development and validation of a fluorescence polarization (FP) based assay for high-throughput screening of modulators of the Lin28–pre-let-7g interaction. A library of 2768 pharmacologically active small molecules, including FDA approved drugs, was screened and several small molecule inhibitors of the interaction between Lin28 and pre-let-7g were identified. Furthermore, two of the active molecules successfully restored Dicer processing of pre-let-7g in the presence of the inhibitor, Lin28, validating the overall approach.

## Materials and methods

### Fluorescence polarisation (FP) measurements

All FP measurements were carried out in a 384-well, low-volume, black, round-bottom polystyrene non-binding surface (NBS) micro-plate (Corning) using a PHERAstar Plus (BMG LABTECH) device. The plate reader was set in polarisation mode with 485 nm and 520 nm excitation and emission filters, respectively. Polarisation was measured and displayed in millipolarisation units (mP). The gain was adjusted for channel 1 and 2 using fluorescein (1 μM) in 50 mM Tris pH 7.5 such that an mP value of ∼35 was obtained.

### Small molecule preparation

Small molecules were obtained from Sigma Aldrich (LOPAC1280 library), the National Cancer Institute (NCI, NCI diversity set II) and an in-house library of the Prof. Shankar Balasubramanian laboratory. The small molecules were prepared at a concentration of 2 mM in dimethyl sulfoxide (100% DMSO) and aliquoted into 384 Well Clear Round Bottom storage plates (Corning). The plates were stored at –80 °C before and between uses. Prior to use in the screening protocol the small molecules were diluted to 100 μM in 1× binding buffer (50 mM Tris pH 7.5, 100 mM NaCl, 1 mM MgCl_2_).

### Small molecule screening protocol

All solutions were dispensed using a Biomek® FXp liquid dispensing robot (Beckman Coulter). Wells were defined as follows: negative control (FAUtpre-let-7g/Lin28), samples (FAUtpre-let-7g/Lin28/small molecules), positive control 1 (FAUtpre-let-7g/Lin28/unlabeled tpre-let-7g) and positive control 2 (FAUtpre-let-7g). A solution of pre-mixed recombinant human Lin28 (0.300 μM) and FAUtpre-let-7g (fluorescein modified tpre-let-7g) (0.017 μM) in 1× binding buffer was distributed to sample, negative control, and positive control 1 well. FAUtpre-let-7g alone (0.017 μM) was added to the positive control 2 well. Small molecules (100 μM, 1× binding buffer, 5 vol% DMSO) were added to the sample wells and binding buffer/DMSO (1× binding buffer, 5 vol% DMSO) was added to the positive control 2 wells. Unlabeled tpre-let-7g (5 vol% DMSO, 1× binding buffer) was added to positive control 1 wells and used as a positive control competitor. The final concentrations of small molecule and unlabeled tpre-let-7g were 20 μM and 0.170 μM, respectively. DMSO was present at 1 vol% in all wells. Total volume of each reaction well was 20 μL. Screening plates were incubated for 20 min at room temperature (RT) prior to FP measurement. FP measurements were taken at 5 min intervals over 25 min. A hit was defined as a small molecule that reduced the change in FP between the negative and positive controls by 50%. All primary hits were repeated in triplicate. Reproducible primary hits were referred to as secondary hits. Conditions described above were applied in all assay development steps, with substitutions of Lin28 for GST, FAUtpre-let-7g for fluorescein and unlabeled tpre-let-7g for alternative RNAs (C/A pre-let-7g mutant and total yeast RNA).

### Electrophoretic mobility shift assay (EMSA)

The secondary hits identified were tested against the full-length pre-let-7g–Lin28 interaction in an EMSA. Small molecules (100 μM, 1× binding buffer, 5 vol% DMSO), binding buffer/DMSO (1× binding buffer, 5 vol% DMSO) or unlabeled pre-let-7g (1× binding buffer, 5 vol% DMSO) were mixed with ^32^P-pre-let-7g in 1× binding buffer and incubated at RT for 30 min. LIN28 (1.5 μM, 1× binding buffer) was added to the negative control (^32^P-pre-let-7g), samples (^32^P-pre-let-7g/small molecules) and positive control 1 (^32^P-pre-let-7g/unlabeled pre-let-7g). The final concentrations in 1× binding buffer were as follows: LIN28 (0.300 μM), small molecules (20 μM) and unlabeled tpre-let-7g (0.170 μM). DMSO was present at 1 vol% in all reactions. Total volume of each reaction well was 20 μL. All mixtures were incubated at RT for 45 min. Glycerol (2.5 vol%) was added to each mixture and protein/RNA band shifts were observed by non-denaturing PAGE and visualized by phosphorimager. Band intensities were quantified using ImageQuant™ software (GE Healthcare) and used to calculate the average proportion of complex formed.

### Dicer processing assay

*In vitro* Dicer processing reactions were performed in a similar manner to that described previously.^9^ The Dicer cleavage reaction and non-cleaved control consisted of ^32^P-pre-let-7g, 1× Dicer buffer (75 mM NaCl, 20 mM Tris-HCl pH 7.5, 3 mM MgCl_2_) and in the case of the former recombinant Dicer (0.1 units, Invitrogen). For the LIN28 inhibition assay, ^32^P-pre-let-7g was pre-incubated with either the corresponding small molecule (1 vol% DMSO) or DMSO alone (1 vol%) in 1× Dicer buffer at RT for 30 min. To these two resulting solutions Lin28 was added and incubated at RT for 45 min. On addition of recombinant Dicer, the reaction mixtures were heated at 37 °C for 5 min. The final concentrations in 1× Dicer buffer are as follows: LIN28 (0.350 μM) and small molecules (20 μM). DMSO was present at 1 vol% in all reactions. Digested products were resolved by denaturing PAGE sequencing gel and visualized by phosphorimager. Cleavage bands were quantified using ImageQuant™ software (GE Healthcare). For quantification of the data ‘relative Dicer processing efficiency’ was used, which is defined as the product intensity divided by total intensity (products and full-length substrate).

### Statistical analysis

For *K*_d_ determination, samples were prepared as described in **Small molecule screening protocol** with a range of compound concentrations. Data was fitted by prism to a hyperbolic curve, fitting to a Hill1 equation. *Z*-Factors for individual screening plates were calculated as 1 – 3(*σp* + *σn*)/(*μp* – *μn*), where *σ* is the standard deviation, *μ* is the mean, *p* is positive control 1 (FAUtpre-let-7g/unlabeled tpre-let-7g/Lin28), and *n* is negative control (FAUtpre-let-7g/Lin28). IC_50_ values were calculated from multiple-point dose–response curves generated from three replicates, using nonlinear regression curves (PRISM 5.0, GraphPad Software).

## Results

To facilitate the identification of small molecule inhibitors of the interaction between pre-let-7g and Lin28, a fluorescence polarization (FP) based binding assay was first established. The principles of the FP assay derive from the ability of rapidly rotating small ligand-bound fluorophores, excited by plane polarised light, to depolarise the light emitted. Upon increasing the volume of the fluorophore complex (*i.e.* through protein–ligand binding), the fluorophore rotates slower, and as a consequence, a larger proportion of the emitted light remains polarised. Monitoring the change in polarised light emitted from the fluorophore upon ligand–protein complex formation can therefore provide a direct measurement of the fraction of ligand bound to the protein. The polarisation value of a sample, *P* is expressed in millipolarisation units (mP), and is defined as the difference in intensity between emitted light in the polarisation plane (*I*_II_) and in the perpendicular plane (*I*_Γ_), divided by the total intensity of emitted light in both planes (eqn (1)).1
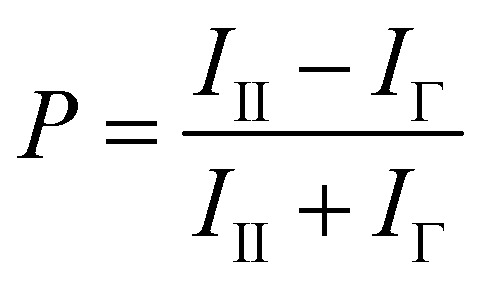



**Fig. 1:**
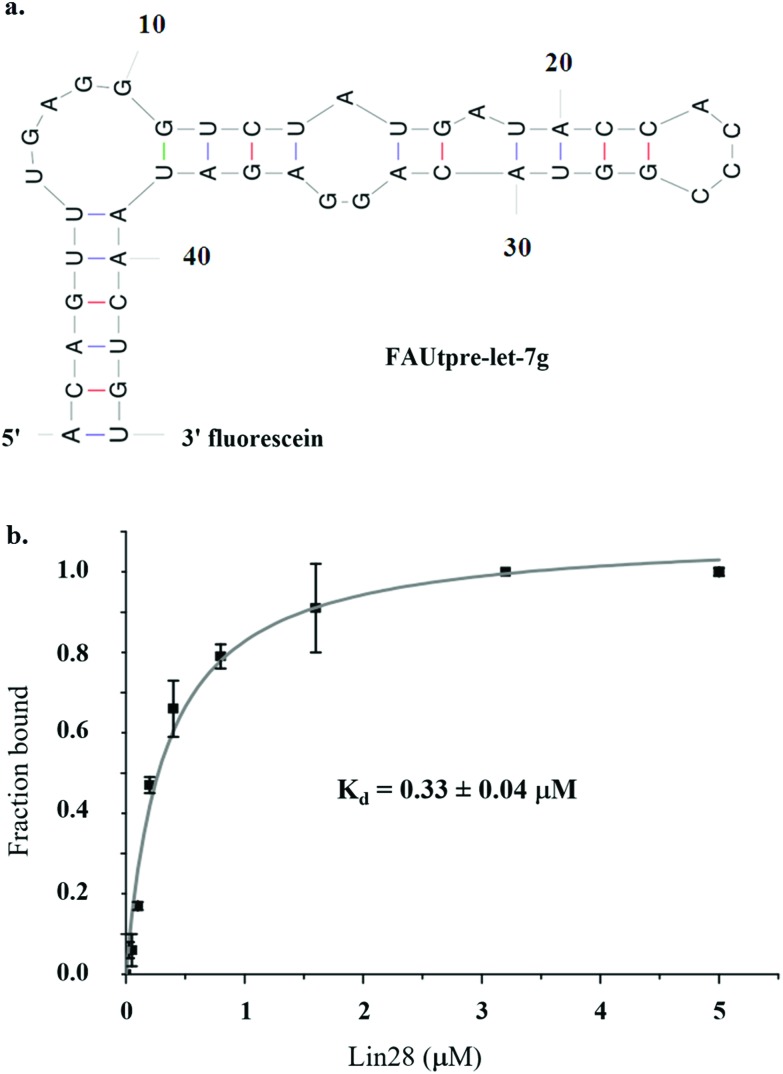
Assay development. a. Structure of FAUtpre-let-7g. b. The change in fluorescence polarisation of 0.017 μM FAUtpre-let-7g in the presence of increasing concentrations of Lin28 was quantified from three independent experiments and represented as the average fraction of Lin28-bound FAUtpre-let-7g. Data was fitted by prism to a hyperbolic curve, fitting to a Hill1 equation and the dissociation constant was calculated (*K*_d_).

As studies to date have implicated the pre-let-7 terminal loop as the main, if not only, docking site for Lin28^9–11,27,36–38^ a truncated version of pre-let-7g (tpre-let-7g), consisting of the pre-let-7g terminal loop with the natural 5′ and 3′ 5 nt flanking regions, was selected as the ligand in this assay. For FP detection, tpre-let-7g was labeled at the 3′ end with fluorescein (FAUtpre-let-7g) (Fig. 1a). The FP of FAUtpre-let-7g was measured in the presence of increasing concentrations of N-terminal glutathione *S*-transferase (GST) tagged-Lin28 (Lin28)^10^ over time. The dissociation constant (*K*_d_) of the FAUtpre-let-7g–Lin28 complex at equilibrium was 0.33 ± 0.04 μM (Fig. 1b). This value is slightly lower than the *K*_d_ value we and others previously obtained using His tagged-Lin28 and GST tagged-Lin28 and tpre-let-7g through gel shift assays in the presence of excess competitor tRNA.^10,36^ To confirm that the change in P of FAUtpre-let-7g observed in the presence of Lin28 (0.33 μM, ΔmP, ∼50 mP) was due to a direct interaction between Lin28 and tpre-let-7g and not due to indirect effects, several control experiments were conducted. The FP signal of FAUtpre-let-7g and fluorescein was not altered in the presence of glutathione *S*-transferase (GST) and Lin28, respectively (ESI Fig. 1a & b†). Furthermore, no significant change in the intensity of FAUtpre-let-7g on addition of Lin28 was detected (ESI Fig. 1c†). Unlabeled tpre-let-7g, used as a specific competitor, successfully depleted the ΔmP. On addition of 1× and 10× unlabeled tpre-let-7g relative to the concentration of FAUtpre-let-7g, ΔmP reduced by ∼50 and 100% respectively (ESI Fig. 2†), confirming that the system is responsive to specific competitors. In contrast, a much larger excess (50×) of a C/A tpre-let-7g mutant (reported to display reduced LIN28 binding more than 8-fold relative to the wild-type^36^) was required to induce a ∼100% reduction in the ΔmP (ESI Fig. 2†). Furthermore, a large excess of total yeast RNA (1000×), a non-specific competitor, was also required to induce a ∼100% reduction in the ΔmP (ESI Fig. 2†). This data confirms that the ΔmP observed is due to specific interactions between tpre-let-7g and Lin28. As DMSO would be used to prepare stock solutions of candidate inhibitors, an additional factor that needed to be considered was the effect of DMSO on the ΔmP. DMSO concentrations up to 1% (v/v) had no effect on the ΔmP, however at DMSO concentrations of ≥2% (v/v) significant variations in the ΔmP were observed (ESI Fig. 3†). A final DMSO concentration of 1% (v/v) was selected for screening. The *Z*′ factor^39^ was used to evaluate the quality and robustness of the assay and to assess its suitability for high-throughput screening. A calculated *Z*′ greater than 0.5 was consistently detected, confirming that the assay was robust. Of note, potential stochastic false positive hits were observed, but only to a minor extent (2%). As standard in drug discovery protocols, identification of such stochastic false positives through hit repetition was crucial to reduce the number of false positives prior to the hit validation stages.

**Fig. 2:**
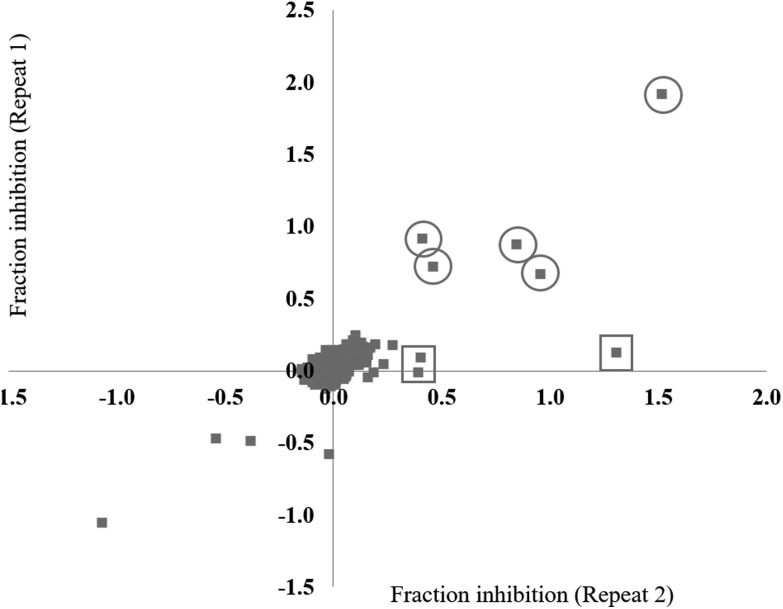
Test screen. The fraction inhibitions observed for each small molecule from repeat 1 and repeat 2 of a test screen of 280 small molecules in the fluorescence polarization assay. The five most highly reproducible hits (≥50% inhibition) are circled. Likely false positives are in squares.

The small molecule library to be tested was obtained from two main sources; the Sigma LOPAC1280 library (1280 compounds) and the NCI diversity set II (1356 compounds). In addition, a small subset of ligands (132 compounds) designed to specifically target nucleic acid structures (laboratory of Prof. Shankar Balasubramanian) were also included in the study, bringing the total number of small molecules to 2768. To validate the assay for high throughput screening (HTS), a test screen was performed using a 280 compound sub-set of the library, each at a final concentration of 20 μM. The data from repeat 1 and repeat 2 were each converted to fraction inhibition values relative to the positive and negative controls. To investigate the reproducibility of the small molecule screening platform, the data from repeat 1 was plotted against the data from repeat 2 (Fig. 2). A hit was defined as a compound that decreased the ΔmP by ≥50%. The majority of the compounds were reproducibly inactive as demonstrated by the large number of points clustered around the zero inhibition value. Five hits were shown to be reproducible (Fig. 2, circles). The irreproducible hits (Fig. 2, squares) are likely stochastic false positives, as noted earlier during the assay development stage. The preliminary small molecule screen had a 1.8% hit rate, which is in the upper range of standard HTS hit rates.^40,41^ The conditions of the assay were adequate for high-throughput small molecule screening.

**Fig. 3:**
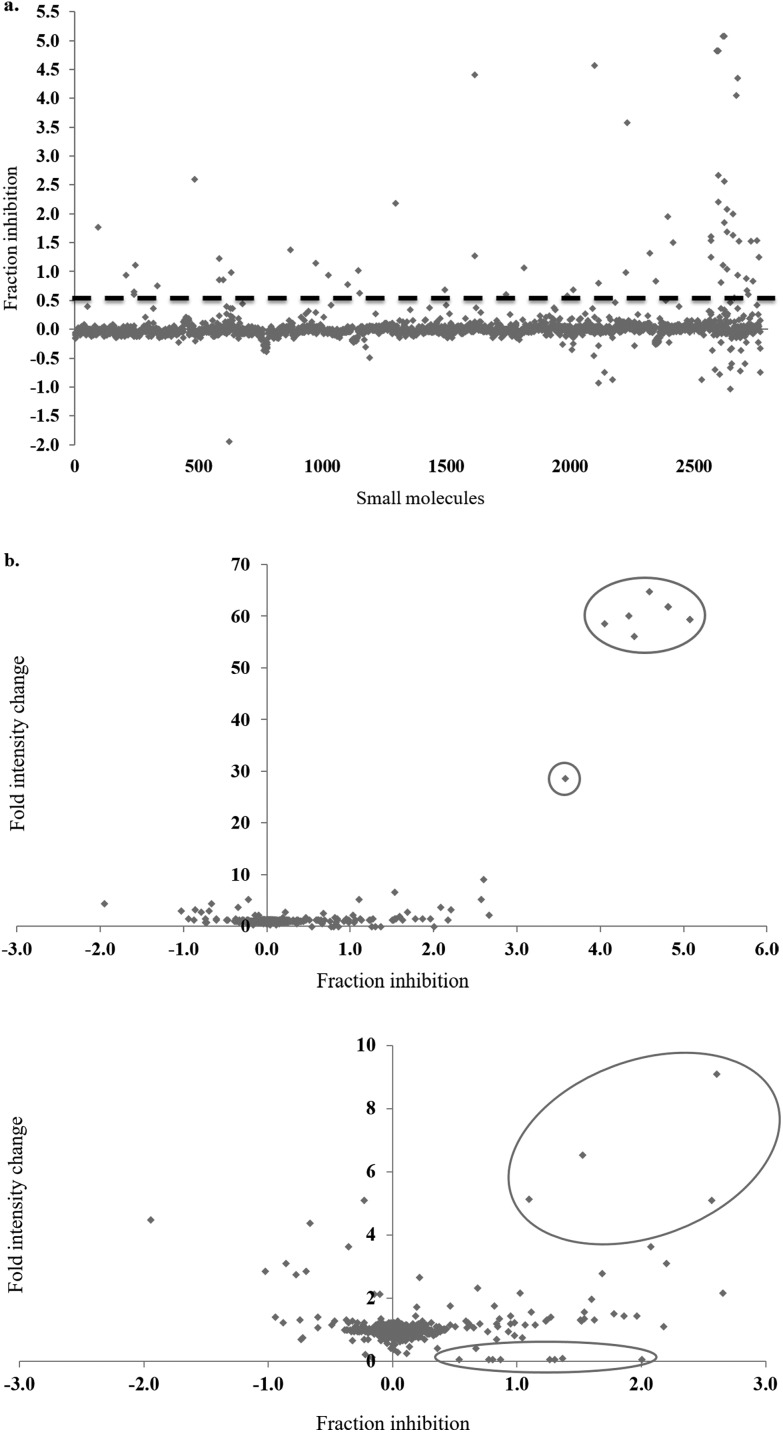
Full small molecule screen. a. The fraction inhibition observed for each compound from a full screen of 2768 compounds in the fluorescence polarization assay. Primary hits are located above the dashed line (>50% inhibition). b. A plot of the fold intensity change (normalised to the control wells) *versus* the fraction inhibition for each compound. Potential false positives are circled. Expanded image below.

**Fig. 4:**
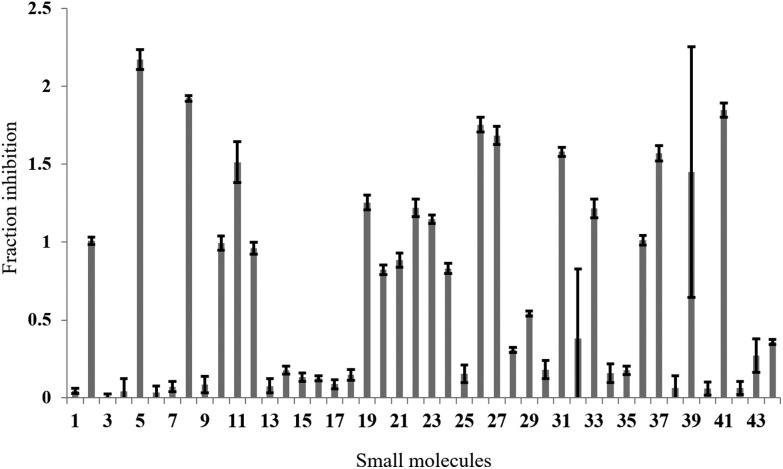
Hit repetition. The average fraction inhibition observed on repetition of 44 primary hits in the fluorescence polarization assay. A secondary hit was defined as a small molecule that reproducibly displayed a fraction inhibition value greater than 0.5.

In the full-scale screen, 2768 small molecules were tested once at a final concentration of 20 μM (Fig. 3a). As found in the test screen, the majority of the small molecules were inactive as demonstrated by the large number of points clustered at the zero inhibition region. 64 primary hits were identified from the full screen, which equates to a 2.3% hit rate. Small molecules can alter the polarisation value without inhibiting the FAUtpre-let-7g–Lin28 interaction. Common causes of such false positives are the use of intrinsically fluorescent compounds, moieties that induce static or dynamic fluorophore quenching, as well as light scattering due to compound precipitation. Several small molecules increased the polarisation above that of the negative control. This resulted in a fraction inhibition that was lower than zero (Fig. 3a), and suggests that the molecules are causing complex aggregation, hence increasing its molecular weight or that the compound was precipitating out of solution (compound precipitation produces scattered light which is highly polarised). Furthermore, a number of small molecules in this screen reduced the polarisation to a value lower than that observed for the positive control (Fig. 3a). This resulted in a calculated fraction inhibition that was greater than 1 and suggested that these small molecules interfere with the fluorescence of the fluorescein label of FAUtpre-let-7g, lowering the baseline polarisation value. Changes in fluorescence upon compound addition can be indicative of the aforementioned false positives. To help identify such false positives, the fold intensity change of each sample well was calculated, and plotted against its fraction inhibition (Fig. 3b). The fold intensity change equates to the total fluorescence intensity of the sample well normalized to the averaged total fluorescence intensity of the control wells. The plot of the fold intensity change *versus* fraction inhibition revealed several likely false positives. These compounds were shown to either increase the total intensity of the well by >5-fold or severely quench the fluorescence to <0.1 (Fig. 3b). These compounds were removed from the primary hits. A hit repetition stage was then applied to assess the reproducibility of the primary hits and in particular, identify stochastic false positives. A total of 44 primary hits were retested and the fraction inhibition displayed by each of these compounds is shown in Fig. 4. A shortlist of 21 small molecules, equivalent to a final hit rate of 0.75%, which is in the range of previously reported values,^40,41^ were shown to reproducibly decrease the ΔmP by ≥50% (secondary hits).

**Fig. 5:**
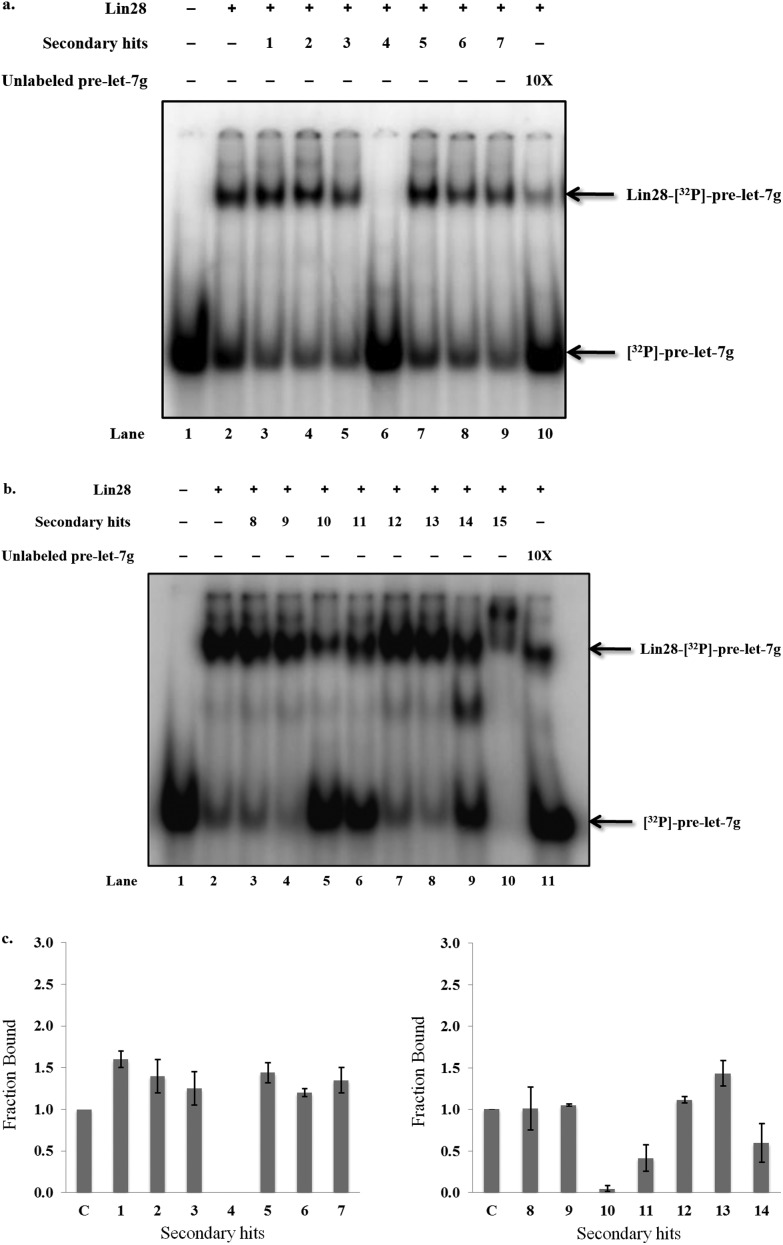
Validation of the secondary hits against the interaction between [^32^P]-pre-let-7g and Lin28 by EMSA. a. & b. Representative EMSAs performed with [^32^P]-pre-let-7g, Lin28 (0.300 μM) and secondary hits (20 μM). Addition of unlabeled pre-let-7g (10×) to the Lin28–[^32^P]-pre-let-7g binding reaction was used as the positive control, and Lin28–[^32^P]-pre-let-7g binding reaction as the negative control. c. Band intensities were quantified using ImageQuant™ and the fraction bound was calculated relative to the signals in the positive and the negative control lanes. C: negative control. Error bars represent the standard deviation of two independent experiments.

**Fig. 6:**
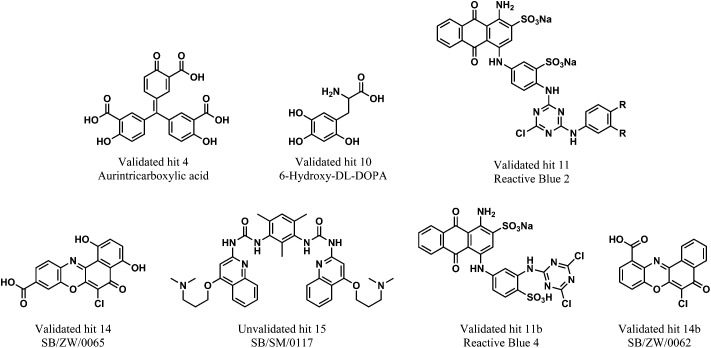
*In vitro* validated inhibitors of the interaction between pre-let-7g and Lin28. The un-validated secondary hit 15 is also shown.

Next, it was crucial to validate the secondary hits against the full-length pre-let-7g, as well as to confirm their activity in an alternative, preferably non-fluorescent, biochemical assay. To fulfil both of these criteria, the secondary hits were tested (at 20 μM concentration) in a radioactivity based EMSA against the interaction between ^32^P-labeled full-length pre-let-7g ([^32^P]-pre-let-7g) and Lin28. Unlike our previous work,^10^ this assay was performed in the absence of competitor RNA; here the lower stoichiometric Lin28–pre-let-7 complexes can be observed.^13^ As several of the secondary hits were from the same family of compounds, one representative compound from each family was chosen for the EMSA validation. Of the 21 secondary hits, a total of 15 were tested and the fraction inhibition for each secondary hit calculated. The attrition rate in the EMSA assay was high (see discussion) with only four compounds confirmed as true positives that inhibit the interaction between [^32^P]-pre-let-7g and Lin28 by ≥50% (Fig. 5a: compare lanes 2 & 10 to lane 6; Fig. 5b: compare lanes 2 & 11 to lanes 5, 6 and 9, Fig. 5c). These compounds, referred to as validated hits 4, 10, 11 and 14, were identified as Aurintricarboxylic acid, 6-hydroxy-dl-DOPA, Reactive Blue 2 and SB/ZW/0065, respectively (Fig. 6). The effect of one secondary hit, SB/SM/0117 (Fig. 6: secondary hit 15) was not detectable *via* this method as the amount of radioactivity and the mobility of the radioactivity through the gel matrix were greatly reduced relative to that of the negative control (Fig. 5b: compare lanes 2 and 10). This suggested that SB/SM/0117 was precipitating out of solution, and this hit was therefore removed from the validated hit collection.

For additional studies, the validated hits were re-purchased or re-synthesised. All validated hits were again shown to inhibit the interaction between Lin28 and pre-let-7g at a concentration of 20 μM in the FP assay. As Reactive Blue 2 is no longer commercially available, a closely related analogue that contained a very similar core structure to Reactive Blue 2, but lacked the aniline substituent on the triazine was purchased. The replacement was referred to as 11b (Fig. 6). The half maximal inhibitory concentration (IC_50_) value for each validated hit was calculated from a plot of concentration *versus* Δmp. All validated hits displayed a dose-dependent inhibition of the Lin28–tpre-let-7 interaction. The IC_50_ values calculated for aurintricarboxylic acid, 6-hydroxy-dl-DOPA, Reactive Blue 4 and SB/ZW/0065 were 1.18 ± 0.23 μM, 7.05 ± 0.13 μM, 10.75 ± 0.1 μM and 4.71 ± 0.16 μM, respectively (ESI Fig. 4†). Compounds SB/ZW/0065, aurintricarboxylic acid, 6-hydroxy-dl-DOPA and Reactive Blue 4 were carried forward to the *in vitro* based functional validation assay. In addition, SB/ZW/0062, a closely related analogue of SB/ZW/0065, which was also identified as a secondary hit but not tested in the EMSA, was also taken forward (Fig. 6, referred to as 14b).

**Fig. 7:**
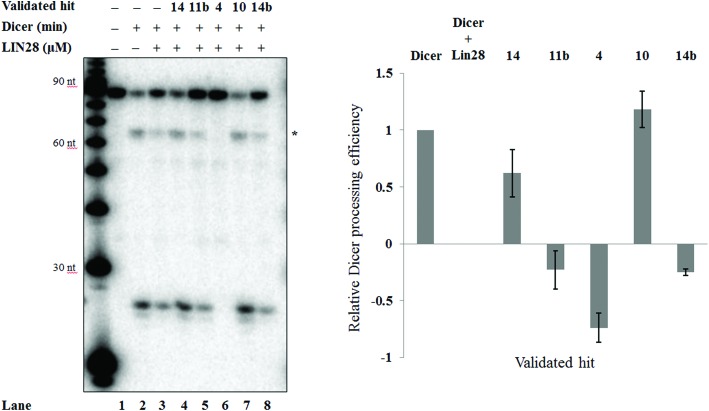
Activity of the validated hits against the Lin28-mediated inhibition of pre-let-7g processing by Dicer. a. Representative autoradiogram of the Dicer processing assay of [^32^P]-pre-let-7g in the presence of Lin28 and the validated hits (20 μM). *: Initial Dicer cleavage product (single cleavage). b. Relative Dicer processing efficiency. Results were normalized relative to the Dicer processing efficiency obtained for [^32^P]-pre-let-7g alone (positive control, lane 1) and in the presence of Lin28 (negative control, lane 2). Error bars represent the standard deviation of two independent experiments.

To assess whether the validated hits could prevent the Lin28-mediated inhibition of let-7g biogenesis, the effect of these compounds on Lin28 blockage of pre-let-7g cleavage by Dicer was assessed through an *in vitro* Dicer processing assay.^10^ In the presence of Dicer we observed a reduction in the amount of pre-let-7g, and the appearance of an approximately 20 nt band, corresponding to the mature let-7g (Fig. 7a, compare lane 1 and lane 2). Upon addition of Lin28, the intensity of the mature let-7g band reduced, and that of the pre-let-7g band increased (Fig. 7a, compare lane 2 & lane 3, Fig. 7b) confirming Lin28 inhibition of Dicer processing of pre-let-7g *in vitro*. The effect of the validated hits upon this Lin28-mediated block in pre-let-7g processing varied greatly. It was noteworthy that one of the validated hits, 6-hydroxy-dl-DOPA (validated hit 10), restored Dicer processing of pre-let-7g to the level observed by Dicer alone (Fig. 7a, compare lane 1 to lane 2), in the presence of Lin28 (Fig. 7a, compare lane 2 and 3 to lane 7, Fig. 7b). In addition, a second compound, SB/ZW/0065 (validated hit 14), also partially restored Dicer processing, in the presence of Lin28 (Fig. 7a, compare lanes 2 and 3 to lane 4, Fig. 7b). However, in contrast, aurintricarboxylic acid (validated hit 4) and, to a lesser degree, SB/ZW/0062 (validated hit 14b) and Reactive Blue 4 (validated hit 11b) inhibited Dicer processing beyond that observed in the presence of Lin28 (Fig. 7a, compare lanes 2 and 3 to lanes 5, 6 & 8, Fig. 7b). For 6-hydroxy-dl-DOPA (validated hit 10), the observed enhance in Dicer processing in the presence of Lin28 was dose dependent (ESI Fig. 5a†) and was not observed in the absence of Lin28 (ESI Fig. 5b†). This confirms that the increase in Dicer processing of pre-let-7g by 6-hydroxy-dl-DOPA is due to inhibition of the Lin28–pre-let-7g interaction.

## Discussion

A fluorescence polarization based *in vitro* assay was established and exploited to identify small molecules capable of inhibiting the direct interaction between Lin28 and a truncated form of pre-let-7g (tpre-let-7g). Using this approach, a library of 2768 pharmacologically active small molecules (including FDA approved drugs) was screened and molecules that successfully prevented binding of Lin28 to tpre-let-7g were revealed. Several of these molecules were subsequently validated as inhibitors of the interaction between Lin28 and full-length pre-let-7g in an alternate biochemical assay. Remarkably, two of the active entities also prevented the Lin28-mediated inhibition of Dicer processing of pre-let-7g *in vitro*, validating the screening approach. These two promising compounds were the dopamine precursor, 6-hydroxy-dl-DOPA and the benzo[*a*]phenoxazine derivative SB/ZW/0065, a novel compound synthesized in the Balasubramanian laboratory.^42^ Of interest, numerous structural analogues of SB/ZW/0065 (12) and 6-hydroxy-dl-DOPA (five) were inactive in the FP screen (ESI Fig. 6 & 7†), suggesting that specific interactions independent from their shared structural scaffold are crucial for their activity. Interestingly, oxidopamine hydrochloride, an untested analogue of 6-hydroxy-dl-DOPA, has been previously identified as a potent small molecule inhibitor of the loading of miRNAs into the RISC complex in cells. Furthermore aurintricarboxylic acid, a known inhibitor of RNA–protein interactions, which profoundly inhibited Dicer in our study, was also active in this study.^43^ Of note, no change in thermal melting and/or RNase foot-prints of pre-let-7g was observed in the presence of 6-hydroxy-dl-DOPA and SB/ZW/0065 at compound concentrations of up to 30 μM, suggesting that these compounds are not binding directly to pre-let-7g (data not shown). The effects of 6-hydroxy-dl-DOPA and SB/ZW/0065 on let-7g levels in Lin28-expressing P19 embryonal carcinoma cells were also assessed. SB/ZW/0065 and 6-hydroxy-dl-DOPA had no significant effect on let-7g levels in this cell system (data not shown).

The attrition rate in the EMSA was unexpectedly high. On evaluation of the FP fraction inhibition and background intensities of the 15 secondary hits, all four validated hits displayed a fraction inhibition between 0.5 and 1.1 and additionally altered the background fluorescence intensity ≤2-fold. Conversely, the majority of the remaining secondary hits (82%) displayed fraction inhibitions >1.1 and/or altered the background fluorescence intensity >2-fold. This suggests that the criteria applied to select hits for EMSA validation (fraction inhibition ≥0.5; background intensity change ≤5-fold) were not sufficiently stringent. For future screening applications a more focused selection process should be considered.

In conclusion, we have identified inhibitors of the interaction between Lin28 and pre-let-7g. Two small molecules capable of inhibiting the interaction between Lin28 and pre-let-7g, and consequently able to restore Dicer-mediated cleavage of pre-let-7g in the presence of the inhibitor Lin28, were found. This study provides biophysical and biochemical proof of concept for the small molecule enhancement of Dicer processing of pre-let-7g. Furthermore, it presents an alternate screening approach to those recently reported by Roos *et al.* ^44^ and Lin *et al.* ^45^ for identification of small molecule inhibitors of the Lin28–pre-let-7-TUTase system. The overall design of this study could be utilized as a basis to identify small molecule inhibitors of this interaction (inclusive of other members of the let-7 family), or other RNA targets of Lin28.
