# Supplementary material for: Identification of small molecule inhibitors of the Lin28-mediated blockage of pre-let-7g processing
Source: Org Biomol Chem. 2016 Oct 4;14(43):10208–16. doi: 10.1039/c6ob01945e (PMC5433426; doi:10.1039/c6ob01945e)
Supplement: Supplementary file 1 [file OB-014-C6OB01945E-s001.pdf]

Electronic Supplementary Material (ESI) for Organic & Biomolecular Chemistry.
This journal is © The Royal Society of Chemistry 2016

Identification of small molecule inhibitors of the Lin28-mediated blockage of pre-let-7g processing

Helen L. Lightfoot[1], *Eric A. Miska**[2] *and Shankar Balasubramanian**[1,3]

[1] Department of Chemistry, University of Cambridge, Lensfield Road, Cambridge, CB2 1EW, UK.

[2] Wellcome Trust Cancer Research UK Gurdon Institute, University of Cambridge, Tennis Court Rd, Cambridge, CB2 1QN, UK.

[3] Cancer Research UK, Cambridge Institute, Li Ka Shing Centre, Robinson Way, Cambridge, CB2 0RE, UK.

To whom correspondence should be addressed. Shankar Balasubramanian (S.B.: telephone, +44 1223 336347; fax, +44 1223 336913; e-mail, sb10031@cam.ac.uk) and Eric Miska (E.A.M.: telephone, +44 1223 767220; fax, +44 1223 334088; e-mail, eric.miska@gurdon.cam.ac.uk).

Key words: Lin28, let-7 miRNA, small molecules, high throughput screening

a.

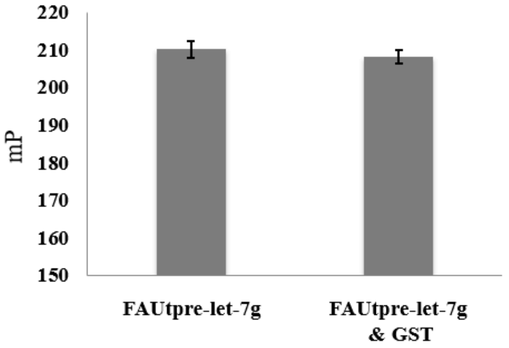

b.

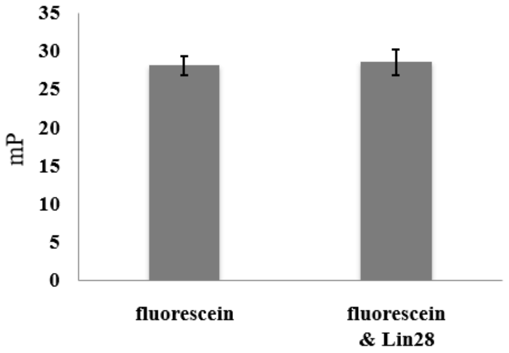

c.

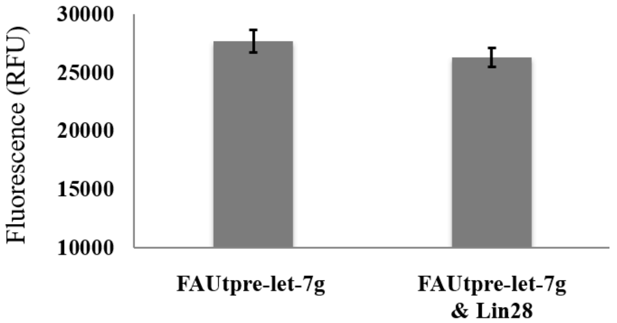

Supplementary Figure 1: Assay development. Fluorescence polarisation of **a**. FAUtpre-let-7g in the presence and absence of GST (0.300 μM) and **b**. Fluorescein in the presence and absence of Lin28 (0.300 μM). **c**. Intensity of FAUtpre-let-7g in the presence and absence of Lin28 (0.300 μM). Data shown was taken at $t = 40$ min and is representative of measurements taken every 20 min for 6 h. Error bars represent the standard deviation of three independent experiments.

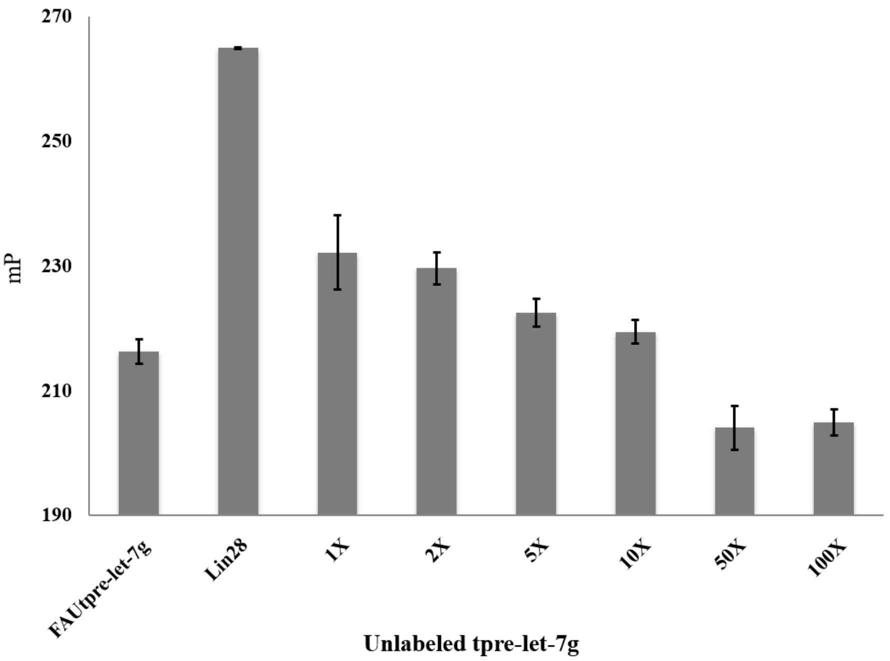

270
250
230
210
190
mP
FAUtpre-let-7g
Lin28
1X
2X
5X
10X
50X
100X
Unlabeled tpre-let-7g

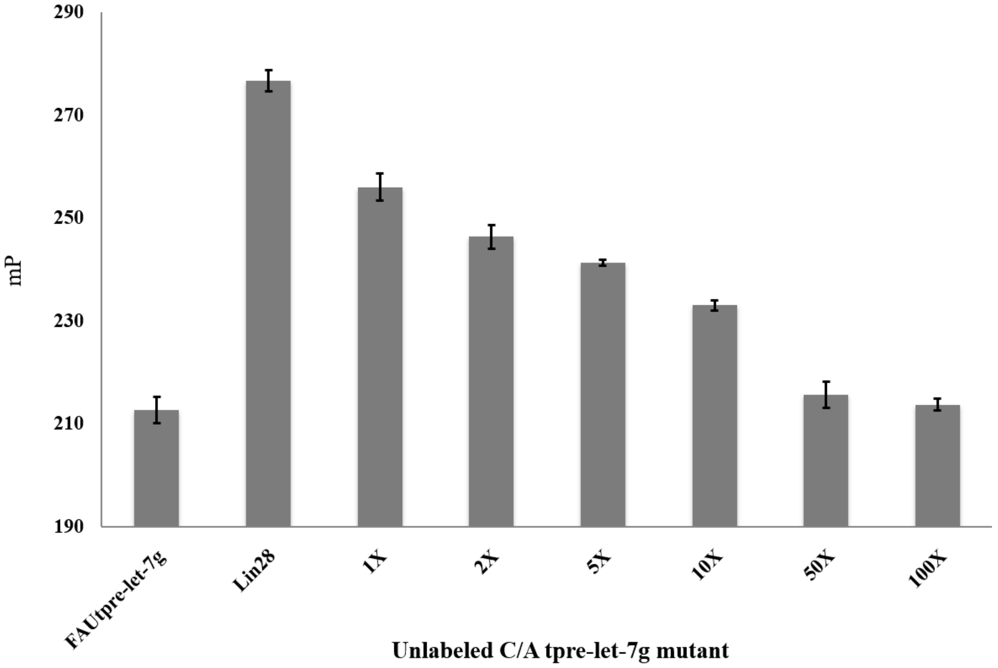

290
270
250
230
210
190
mP
FAUtpre-let-7g
Lin28
1X
2X
5X
10X
50X
100X
Unlabeled C/A tpre-let-7g mutant

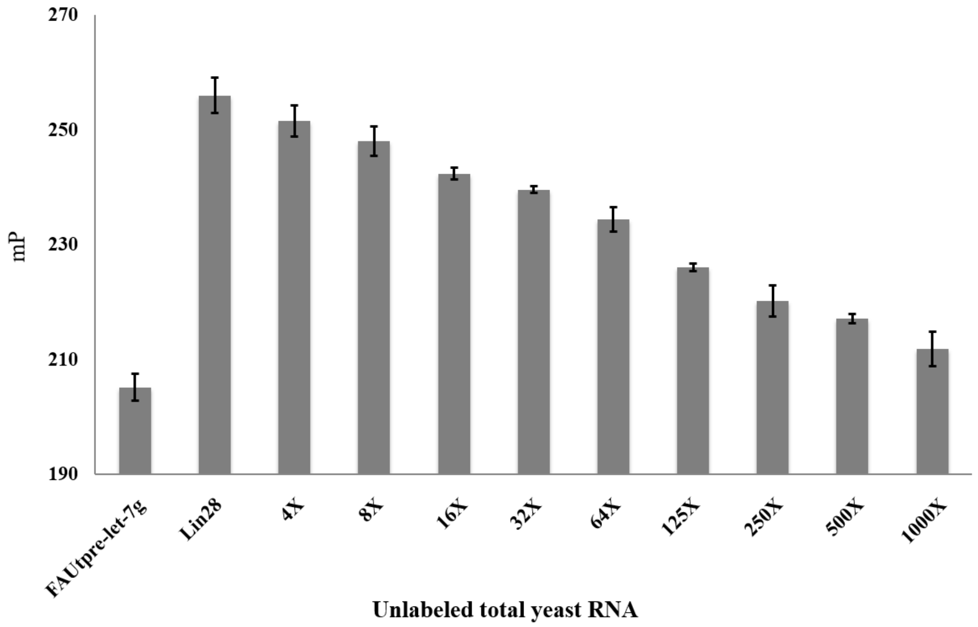

Supplementary Figure 2: Assay development. The effect of unlabeled competitors, tpre-let-7g (specific), C/A tpre-let-7g mutant and total yeast RNA (non-specific), on fluorescence polarisation of FAUtpre-let-7g in the presence of Lin28. Competitor concentrations represented as the amount of competitor relative to FAUtpre-let-7g. Error bars represent the standard deviation of at least two independent experiments.

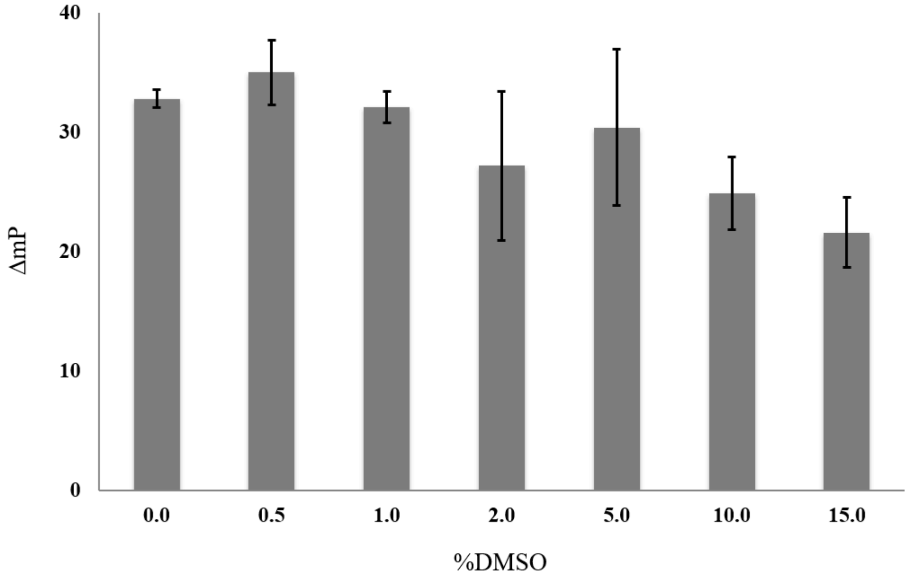

Supplementary Figure 3: Assay development. Effect of DMSO on the change in fluorescence polarisation of FAUtpre-let-7g in the presence of Lin28. Error bars represent the standard deviation of three independent experiments.

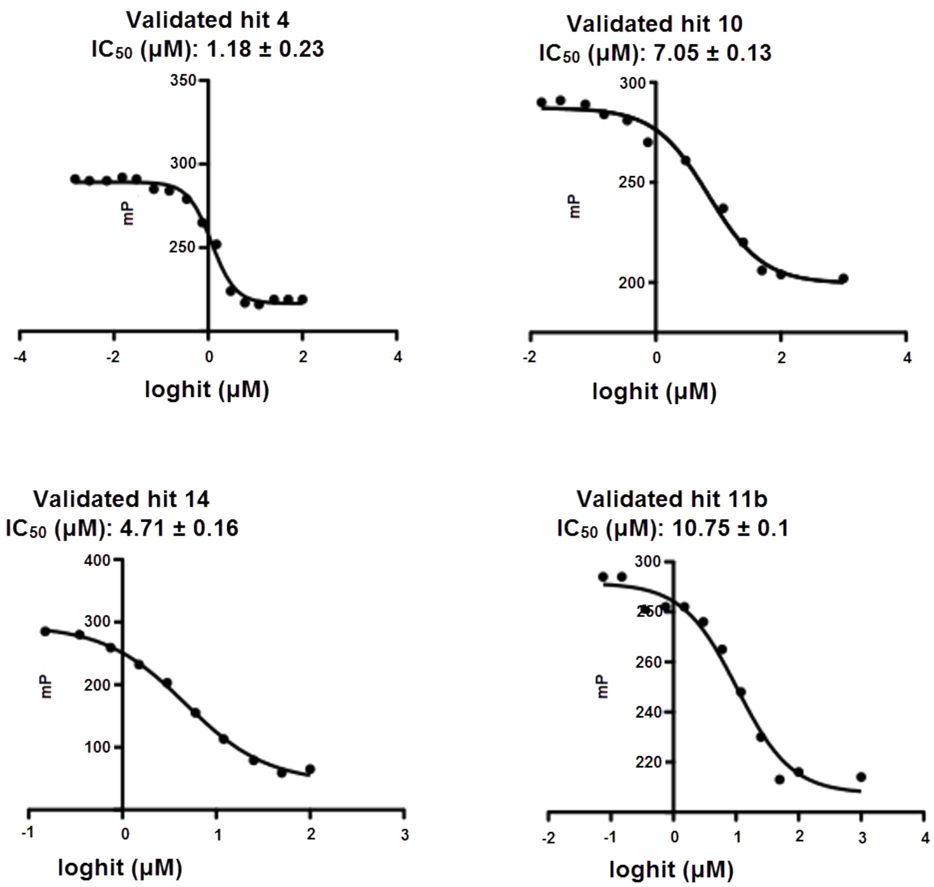

Supplementary Figure 4: Dose response curves for the effect of validated hits on the interaction between FAUtpre-let-7g and Lin28 in the fluorescence polarization assay. The polarisation was measured in three independent experiments. A non-linear curve-fitting program (Prism) was used to fit a dose response curve to a plot of polarisation versus the log of the hit concentration (μM). The IC_{50} values were calculated.

a.

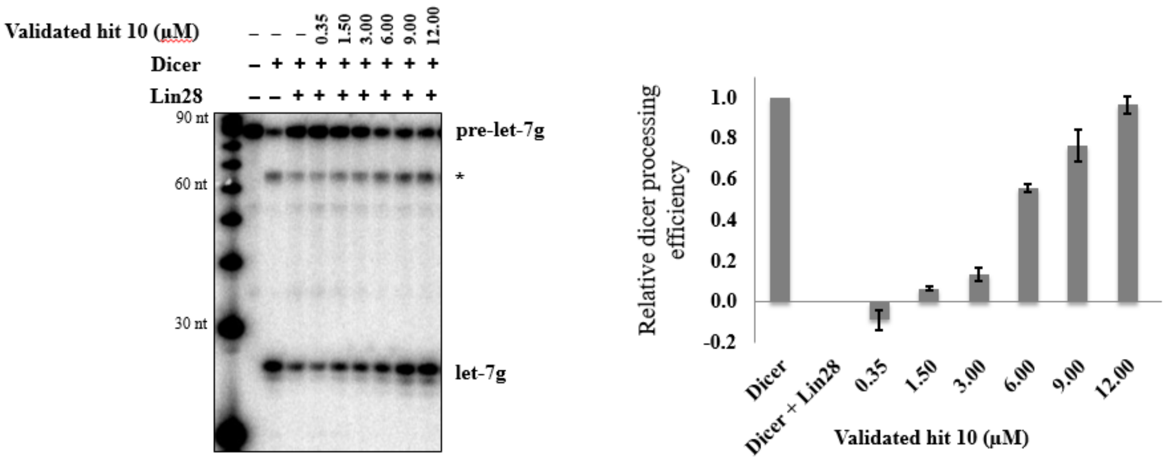

b.

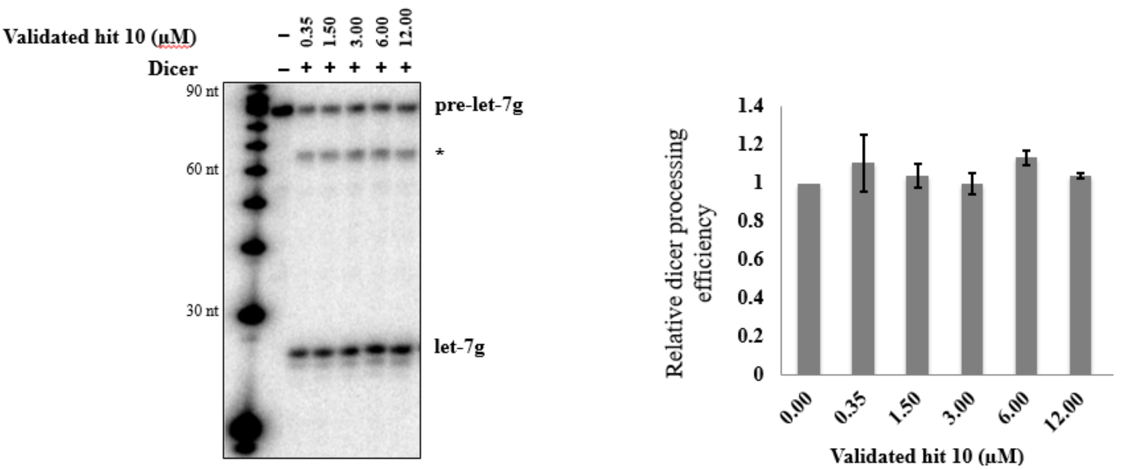

Supplementary Figure 5. Dose dependent effects of 6-hydroxy *DL*-DOPA (validated hit 10) on **a**. Lin28-mediated inhibition of pre-let-7g processing by Dicer and **b**. Dicer processing in the absence of Lin28. The extent of Dicer processing was quantified by calculating the relative Dicer processing efficiency. Results were normalized relative to the Dicer processing efficiency obtained for [^{32}P]-pre-let-7g alone and in the presence of Lin28 (**a**.). * :initial Dicer cleavage product (single cleavage). Error bars represent the standard deviation of two independent experiments.

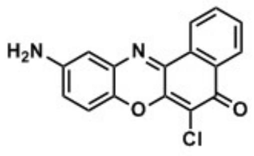

SB/ZW/0144
FI = 0.02

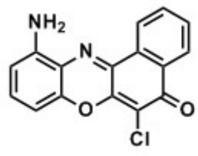

SB/ZW/0145
FI = 0.01

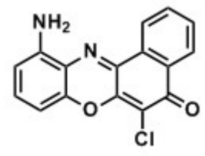

SB/ZW/0142
FI = -0.6

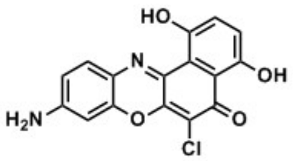

SB/ZW/0141
FI = 0.28

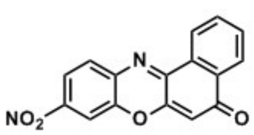

SB/ZW/0059
FI = 0.01

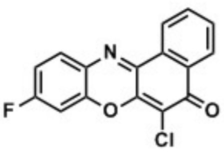

SB/ZW/0060
FI = -0.23

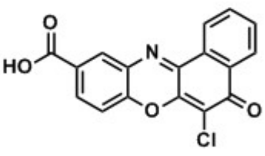

SB/ZW/0061
FI = 0.82
FI2 = 0.27

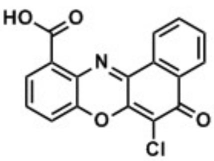

SB/ZW/0062
FI = 1.69
FI2 = 1.1

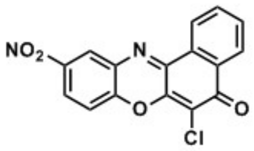

SB/ZW/0063
FI = 0.24

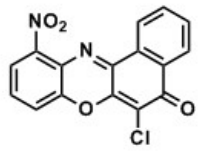

SB/ZW/0064
FI = -0.07

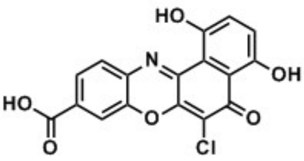

SB/ZW/0065
FI = 1.05
FI2 = 1.21

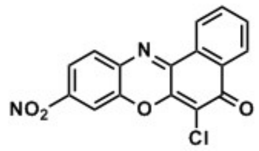

SB/ZW/0031
FI = -0.07

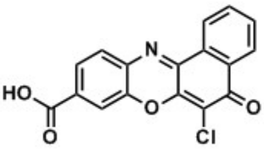

SB/ZW/0032
FI = 0.2

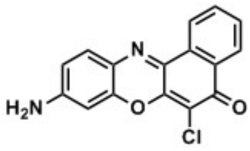

SB/ZW/0033
FI = -0.02

Supplementary Figure 6: Analogues of validated hit 14 (SB/ZW/0065) from the primary FP screen. FI= Fraction inhibition (FP) from primary screen. FI2= Fraction inhibition (FP) from secondary screen.

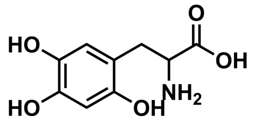

6-Hydroxy-*DL*-DOPA
FI = 0.98
FI2 = 0.99

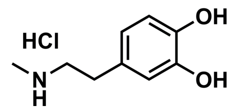

N-Methyldopamine hydrochloride
FI=-0.06

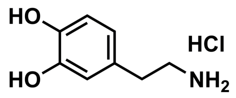

Dopamine hydrochloride
FI=-0.04

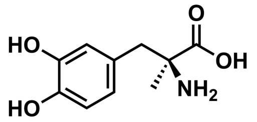

L-alpha-Methyl DOPA
FI=-0.03

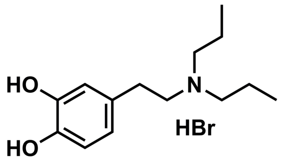

Dipropyldopamine hydrobromide
FI=-0.07

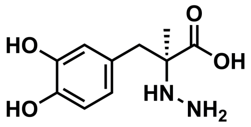

S-(-)-Carbidopa
FI= -0.12

Supplementary Figure 7: Analogues of validated hit 10 (6-Hydroxy-DL-DOPA) from the primary FP screen. FI= Fraction inhibition (FP). FI2= Fraction inhibition (FP) from secondary screen.
